# Imaging latent tuberculosis infection with radiolabeled nitroimidazoles

**DOI:** 10.1007/s40336-016-0166-y

**Published:** 2016-03-22

**Authors:** Alfred O. Ankrah, Andor W. J. M. Glaudemans, Mike M. Sathekge, Hans C. Klein

**Affiliations:** Department of Nuclear Medicine and Molecular Imaging, University Medical Centre Groningen, University of Groningen, Hanzeplein 1, 9700 RB Groningen, The Netherlands; Department of Nuclear Medicine, University of Pretoria, Pretoria, South Africa; Department of Psychiatry, University Medical Centre Groningen, University of Groningen, Groningen, The Netherlands

In a previous paper, an overview of the use of PET/CT in the management of tuberculosis (TB) was provided and the potential role of nitroimidazole imaging in LTBI was considered [1]. During latent TB, dormant bacilli putatively reside in a hypoxic environment of caseating lung granulomas. Exposure of Mycobacterium tuberculosis (*Mtb)* to progressive hypoxia in vitro induces a dormant state characterized by reduced replication and metabolism analogous to that postulated for bacilli in LTBI. In vitro, *Mtb* has been shown to enter microaerophilic nonreplicating persistent (NRP) stage 1, if the dissolved oxygen content falls below 1 %. NRP stage 1 is characterized by thickening of the outer cell wall of Mtb and termination of DNA synthesis. If the oxygen content further drops to approximately 0.06 %, the bacilli enter NRP stage 2, which is accompanied by reduced susceptibility to standard anti-TB drugs but increased susceptibility to nitroimidazole drugs [2]. In light of this, metronidazole, an antimicrobial agent active against anaerobic bacteria, has been investigated as a possible therapy for TB, particularly latent and persistent TB. Metronidazole has recently been documented to be a viable option for treatment of multidrug-resistant (MDR) TB [3].

Oxygen is an essential nutrient for mammalian cells, because of its role as the terminal acceptor in oxidative phosphorylation. When tissues are gradually deprived by oxygen over time, the cells adapt to the new physiological state of insufficient oxygen by upregulation and transcription of certain proteins to meet metabolic demands. This phenomenon is called hypoxia and it may occur in diseases such as stroke, myocardial infarction with sudden vascular occlusion or as a consequence of poor perfusion, e.g., in diabetic limbs, arthritic joints or infections such as tuberculosis. Clearly, the ability to identify hypoxia has implications in a wide range of medical conditions. A clinically suitable method for detection of hypoxia is thus essential. In oncology, invasive probe-based methods have been previously used including the Eppendorf needle electrode and optical needle probes such as OxyLite. Alternatively, immunohistochemical methods have been applied to detect markers of hypoxia in biopsy samples taken from patients. These markers include both exogenous systemically delivered probes that are administered to the patient, localizing hypoxic regions, and are detected by antibody techniques in tissue specimens, as well as endogenous proteins that are overexpressed under hypoxic conditions. However, the invasiveness of these procedures as well as their susceptibility to sampling error has encouraged the development of image-based methods for detecting and quantifying hypoxia [4]. PET probes have been used to investigate hypoxia in cancer extensively. There is adequate evidence to show that *Mtb* is exposed to hypoxia in the granuloma in latent TB [5]. It can therefore be envisaged that hypoxic granuloma in TB and in anaerobic and microaerophilic infections could be detected by PET probes for hypoxia imaging. The heterogeneous lesions both in active disease and subclinical disease could be studied by combined PET/CT imaging. The CT component can localize the granulomas and PET can demonstrate the presence or absence of hypoxia within these granulomas or even outside the granulomas, where some latent *Mtb* can be localized.

Molecules containing nitroimidazole have emerged as the most prevalent type of molecular probe for hypoxia imaging. The mechanism for localization within cells has been thoroughly elucidated. Host cellular nitroreductase enzymes reduce the NO_2_ group to the nitro anion radical. This reversible process takes place in all cell types. In normoxic cells, adequate oxygen is present to oxidize the NO_2_ radical back to the original NO_2_ group. Thus, the molecule shuttles between the NO_2_ and NO_2_ radical anion as it circulates through tissues. The lack of oxygen in hypoxic tissues prevents the radical anion from reverting back to the original NO_2_, and the radical anion is stabilized and further reduced to NHOH and subsequently NH_2_. The amine NH_2_ group is reactive and binds to cellular macromolecules, trapping it within the cell. Through this process, the molecule accumulates in hypoxic tissue [6]. Nitroimidazole-based probes are used for hypoxia detection through a number of techniques including PET, single-photon emission tomography (SPECT) and immunohistochemistry.

There are currently a number of 2-nitroimidazole compounds available. These include ^18^F-fluoromisonidazole (^18^F-FMISO), ^18^F-fluoroazomycin arabinoside (^18^F-FAZA), ^18^F-fluoroerythronitroimidazole (^18^F-FETNIM), ^18^F-2-nitroimidazoltri- and pentafluoropropyl acetamide (^18^F-EF3 and ^18^F-FF5), ^18^F-flortanidazole (^18^F-HX4), and [^18^F] (1-(2-1-(1H-methyl) ethoxy)-methyl-2-nitroimidazole (^18^F-RP-170). These tracers have been validated for detection of hypoxia in different cancer types. ^18^F-FMISO, the prototype 2-nitroimidazole, has been extensively studied, and other tracers have been developed to address shortfalls with this tracer, such as its stability, and slow clearance from background tissue causing modest signal to noise ratio. ^18^F-FAZA and ^18^F-FETNIM are more hydrophilic than ^18^F-MISO, thus should have better clearance than ^18^F-MISO. ^18^F-EF3 and ^18^F-EF5 are relatively more lipophilic than ^18^F-MISO but more stable and a study of the metabolites showed the tracers were unchanged.

Although there have been reports of hypoxia playing a role in infectious disease, there are few studies involving hypoxia imaging in infection. ^18^F-FMISO was able to detect anaerobic odontogenic infections with a sensitivity of 93 % and a specificity of 97 % [7]. ^18^F-FAZA has been evaluated in vitro and in vivo to assess alveolar echinococcosis (AE) infection caused by the larval from metacestode of *Echinococcus multilocularis* in rodents [8]. ^18^F-FAZA displayed a slightly elevated uptake in the *Echinococcus multilocularis* vesicles in the in vitro studies, but was not found useful in in vivo studies. This appears to be the first and only study up till now, in which ^18^F-FAZA has been used to evaluate infection. It is important to note that metronidazole which is a nitroimidazole has not been found to be effective against alveolar echinococcosis. Metronidazole is a prodrug that is activated under conditions of hypoxia. Metronidazole is an effective therapy for the parasites *Entamoeba histolytica* and *Giardia lamblia*. Both parasites are found in hypoxic habitats. Both parasites reside in the intestinal lumen, whereas *Entamoeba histolytica* is also present in abscesses. The hypoxic environment provides the right milieu for activation of the nitroimidazole drug. The metacestode of *Echinococcus multilocularis* is not subject to hypoxic conditions as it is transported through blood and settles in organs (usually the liver or brain). It is no surprise, therefore, that metronidazole is neither used nor effective for the treatment of alveolar echinococcosis even though metronidazole has been shown to have different modes of action in different parasites [9]. In the only study that has been conducted with ^18^F-FAZA involving *Echinococcus multilocularis* till now, the uptake was minimal and limited to the periphery of the AE lesions. In the same study, both ^18^F-FDG and ^18^F-FLT showed good uptake, indicating imaging of glycolytic and DNA proliferation of metacestode and that probably there was not enough hypoxia in the AE lesion to be imaged by a nitroimidazole.

PET/CT imaging with radiolabeled nitroimidazoles could provide a unique opportunity to non-invasively study the hypoxic state of all granulomas within the body. In vitro evidence supports the potential use of hypoxia imaging in TB. Pimonidazole is a nitroimidazole used for immunohistochemical staining of hypoxic tissue. The intensity of pimonidazole staining was found to correlate directly with the number of encapsulated living *Mtb* bacteria 28 days after hypoxia was induced in an in vivo model of latent TB. This suggests that radiolabeled nitroimidazole PET/CT imaging may provide information not only on the presence of hypoxia, but also on the number of latent bacilli present in a granuloma [10]. The concept of imaging a microorganism with a drug, the pathogen is susceptible to has already been used in PET/CT imaging for several microorganism including TB [1].

Imaging TB with radiolabeled nitroimidazole could potentially provide answers not only for the latent stage of TB, but may help in assessing risk of progression to reactivation based on the number of dormant or persistent bacilli. It may be instrumental in developing new antimicrobials that would need to be taken for only a short period of time, as these drugs would target the latent phenotype bacteria. Future research may provide new insights into the biology of the hypoxic TB granulomas, and thereby increase our understanding of the role of hypoxia in the latency and persistence stage. In the future, hypoxia imaging may also provide a stratification approach for individuals with LTBI and serve as an objective test in determining individuals with LTBI who would require treatment. Finally, hypoxia imaging may help with the development of new vaccines targeting patients with LTBI or persistent (MDR-TB) and may promote the development of new TB drugs targeting LTBI which will eventually lead to reducing the duration of current treatment. This would increase adherence and ultimately make the goal of eradication of TB by 2050 achievable.
